# Calcium – a scoping review for Nordic Nutrition Recommendations 2023

**DOI:** 10.29219/fnr.v67.10303

**Published:** 2023-12-19

**Authors:** Jóhanna E. Torfadóttir, Kirsti Uusi-Rasi

**Affiliations:** 1 Centre of Public Health Sciences, University of Iceland, Reykjavik, Iceland; 2 Directorate of Health, Reykjavik, Iceland; 3 The UKK Institute for Health Promotion Research, Tampere, Finland

**Keywords:** calcium, minerals, bone, nutrition recommendations

## Abstract

The aim of this scoping review was to conduct evidence-based documentations between calcium (Ca) intake and health outcomes for updating dietary reference values (DRVs) and food-based dietary guidelines (FBDGs) in the sixth edition of Nordic Nutrient Recommendations (NNR2023). The systematic literature search was limited to reviews on human data published between 2011 and June 2021. Systematic reviews (SRs) and original publications of relevance for this scoping review were included. A common practice of designing studies on health outcomes related to Ca supplement intake is to examine combined Ca and vitamin D, and therefore, a combination of Ca with vitamin D (CaD) was included in this review. In total, 27 studies addressing the association between dietary or supplemental Ca on bone health, bone mineral density (BMD), pregnancy-related outcomes, cardiovascular diseases (CVD), cancers, obesity, and mortality were reviewed. SRs showed that both dietary and supplemental Ca intakes were positively associated with BMD, but evidence did not support the benefit in fracture prevention. Current evidence did not support that Ca or CaD supplementation increases risk of coronary heart disease or all-cause mortality in older adults, but that Ca may be beneficial for hypertension, especially in young people. Increasing Ca intake may be beneficial during pregnancy, especially for those at high risk of pre-eclampsia due to ethnicity, age, high BMI, and those with low baseline Ca intake. The associations between high Ca intake and cancers were varied, with strong evidence that high consumption of dairy products is protective against colorectal cancer and limited-suggestive evidence that dairy products and diets high in Ca might also be protective against breast cancer. Moreover, there is limited-suggestive evidence that dairy products and diets high in Ca increase the risk of prostate cancer. Based on current evidence, Ca intake is beneficial or neutral in relation to most of the outcomes evaluated in this review. Data from the Nordic countries show that average Ca intake is around the same as previously recommended by NNR. However, the average Ca intake in the Baltic countries is below the recommendations.

## Popular scientific summary

- Calcium is the most abundant mineral in the body, and over 99% is stored in bones and teeth.
- As well its structural role of the skeleton, calcium is an essential regulator of several body functions, such as muscle contraction, function of the nervous system, and blood clotting.
- The requirement for calcium is currently based on the size of the calcium reserve.
- Dairy products are the largest sources of calcium in Nordic and Baltic countries.
- Convincing evidence that the intake of calcium above 1000 mg per day in healthy adults prevents cardiovascular disease, cancer, obesity, or fractures is lacking.

Calcium (Ca), as most nutrients, is necessary for the optimal function of most body systems. It is the principal cation of bone, and without an adequate intake, it is not possible either to build or maintain a fully normal skeletal mass. Calcium retention, reflective of bone mass, increases linearly with calcium intake below the threshold intake but is unrelated to intake above the threshold intake (1). Calcium supplementation with or without vitamin D supplementation is widely used especially in the elderly and has been shown to modestly reduce the risk of new fragility fractures, particularly in older individuals in residential care (2). Beyond its role in maintaining bone health, sufficient dietary or supplemental calcium intake may be associated with favorable non-skeletal outcomes, such as reduced risk of the development of adenomatous polyps in the colon, cancers, and pre-eclampsia and high blood pressure (BP), although evidences have not always been convincing (3).

Calcium supplementation either alone or in combination with vitamin D was viewed as extremely safe, other than gastrointestinal side effects and a slightly increased risk of renal stones, until research results published by Bolland et al. in 2008 raised some concerns about possible association with increased risk for cardiovascular events (4).

Plant-based diets may have several health-related benefits, but on the other hand, they can lead to low intakes of some nutrients, for example, of calcium and vitamin D, which are essential and, especially, important for bone health. When calcium intake is insufficient for any reason, compensatory loss of calcium from the bone follows. Attention to potential shortfall nutrients through the careful selection of foods or fortified foods, or the use of supplements can help ensure healthy bone status to reduce fracture risk in individuals with suboptimal nutrient intake (5–7).

The aim of this review was to update the recent scientific evidence on requirements and health effects of calcium to update current dietary reference values (DRV) valid in Nordic countries (Box 1). This review focuses on independent effects of calcium from diet and from supplements. However, given that most trials nowadays use calcium in combination with vitamin D, distinguishing between the health outcomes for one nutrient versus the other is challenging. When calcium versus placebo comparisons were not presented, studies on combination of calcium with vitamin D were used.

Box 1 Background papers for Nordic Nutrition Recommendations 2023.

- This paper is one of many scoping reviews commissioned as part of the Nordic Nutrition Recommendations 2023 (NNR2023) project (8).
- The papers are included in the extended NNR2023 report, but, for transparency, these scoping reviews are also published in Food & Nutrition Research.
- The scoping reviews have been peer reviewed by independent experts in the research field according to the standard procedures of the journal.
- The scoping reviews have also been subjected to public consultations (see report to be published by the NNR2023 project).
- The NNR2023 committee has served as the editorial board.
- While these papers are a main fundament, the NNR2023 committee has the sole responsibility for setting dietary reference values in the NNR2023 project.

## Methods

This review was conducted in accordance with the protocol developed within the NNR2023 project (8). The literature search was conducted in PubMed/MEDLINE using a string defined in the search strategy ((“calcium, dietary”[MeSH Terms] AND (“2011”[PDAT]: “3000”[PDAT])) AND review[Publication Type]) AND Humans[Filter]. The search was limited to reviews on human data published from 2011 to October 2019. Titles (and abstracts, where necessary) were scanned for relevance, and potentially relevant sources were retrieved. The search was conducted to June 2021. Qualified systematic reviews (qSR) were identified by inclusion and exclusion criteria described by Arnesen et al. (9, 10). Furthermore, we used snowballing for SRs and original publications, which had remained out of the original search, and they were additionally included. A detailed protocol for performing this review has been described previously (11). All sources of evidence considered in this scoping review adhere to the eligibility criteria determined by the NNR2023 project.

**Fig. 1:**
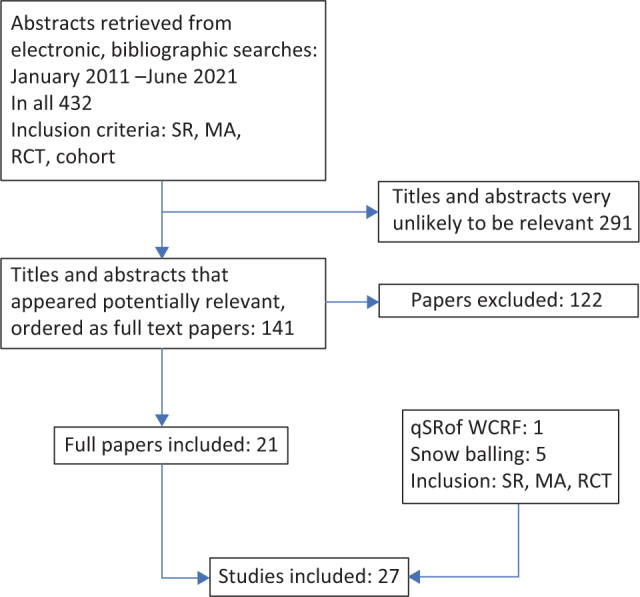
Flow chart.

The PubMed/MEDLINE search in June 2021 resulted in 432 publications. On the grounds of the title, 291 publications were removed, and 141 publications remained for an abstract review. The abstracts of these publications were reviewed; of which, 122 publications were removed, leaving 21 publications for further evaluation. In addition, one qSR was identified for this review (12), namely, the report *Meat, fish and dairy products and the risk of cancer* by the World Cancer Research Fund (WCRF) (13). Also, five snowballs were included (Fig. 1). All reviews with relevant outcomes are presented in Tables 1 to 4.

**Table 1:** Characteristics of the studies evaluating calcium and cardiovascular and related outcomes

Author Year	Outcomes	Type of study	Exposure	Exposure	Conclusion
	CVD				
Chung et al.* Ann Intern Med 2016 (43)	Cardiovascular diseases (CVD) mortality, total stroke, or stroke mortality	SR and MA 4 RCTs, 21 prospective cohort, and nested case–control studies Healthy adults N ranged from 755 to 388,229 Follow-up from 8 to 28 years	Ca supplementation with or without vitamin D Ca intake within tolerable upper intake levels (2,000 to 2,500 mg/day) HR (95% CI) for: CVD 1.01 (0.94 to 1.07) IHD 1.01 (0.95 to 1.08) Stroke mortality1.04 (I 0.96 to 1.14) Total stroke 1.02 (0.94 to 1.10)		Dietary and supplemented Ca intakes are not associated with CVD risks among generally healthy adults within the tolerable upper intake levels (ULs, 2,000 to 3,000 mg/day)
Lewis et al.* J Bone Miner Res 2015 (41)	Coronary heart disease (CHD) risk, including MI, angina pectoris, and acute coronary syndrome, and chronic CHD	MA 18 RCT *N* = 63,563 with 3,390 CHD events and 4,157 deaths	Ca supplementation with or without vitamin D RR (95% CI) for: CHD events (5 RCT) 1.02 (0.96–1.09) All-cause mortality (17 RCT) 0.96 (0.91to 1.02) MI 1.08 (0.92 to 1.26) Angina pectoris and acute coronary syndrome 1.09 (0.95 to 1.24) Chronic CHD 0.92 (0.73 to 1.15)	Ca intake (≥1,000 vs. <1,000 mg/day) HR (95% CI) for: CVD mortality 1.01 (0.94 to 1.07) Stroke mortality 1.04 (0.96 to 1.14) Total stroke 1.02 (0.94 to 1.10)	Current evidence does not support that Ca or CaD supplementation increases CHD or all-cause mortality risk in older women
Asemi et al.* (2015) (44)	All-cause mortality, CVD, and cancer	SR and MA 22 observational studies Aged 8 to >65 years Follow-up from 4.6 to 28 years *N* = 2,346,368 Deaths, *N* = 81,298	Total, dietary, and supplementary Ca intake Total calcium intake RR (95% CI) CVD mortality 1.05 (0.83 to 1.34) All-cause 1.16 (0.83 to 1.64) Cancer 1.49 (0.79 to 2.83) Dietary Ca intake CVD mortality 0.88 (0.78 to 0.99) All-cause 0.84 (0.70 to 1.00) Cancer 0.94 (0.83 to 1.06) Supplemental Ca intake RR (95% CI) CVD mortality 0.95 (0.82 to 1.10) All-cause mortality 0.91 (0.88 to 0.94) Cancer 0.91 (0.88 to 0.94)	For studies with a mean follow-up duration of >10 years. Total Ca intake RR (95% CI) CVD mortality 1.35 (1.09 to 1.68) For studies with a mean follow-up duration of ≤10 years Dietary Ca CVD mortality 0.88 (0.78 to 0.99)	No association between total and dietary Ca intake and CVD, all-cause or cancer mortality In studies with a long follow-up, total calcium intake was associated with increased risk of CVD mortality In studies with a shorter follow-up, dietary calcium intake seemed to protect of all-cause (16%) and CVD (12%) mortality
**Muyng et al.** Nutrients 2021 (42)	CVD, CHD, cerebrovascular	MA 13 RCTs *N* = 14,692 participants in an intervention group and 14,243 participants in a control group	Ca supplements ≥500 mg/day versus placebo In postmenopausal women, RR (95% CI) for: CVD 1.15 (1.06 to 1.25) CHD 1.16 (1.05 to 1.28) Cerebrovascular disease 1.13 (0.97 to 1.31)	In the subgroup meta-analysis, dietary calcium intake of 700–1,000 mg per day or supplementary calcium intake of 1,000 mg per day significantly increased the risk of CVD and CHD	Ca supplements increased a risk of CVD by about 15% in healthy postmenopausal women
	Obesity				
Booth et al.* Br J Nutr 2015 (36)	Body weight and body composition	SR, MA 41 including 51 trial arms; 31 with dairy foods (*N* = 2,091), 20 with Ca supplements (*N* = 2,711) Adults >18 years of age Intervention ≥12 weeks	Ca supplementation and increased dairy foods	Ca supplementation Mean change (95% CI) Body weight -0.17 (-0.70 to 0.37) kg Body fat -0.19 (-0.51 to 0.13) kg	No evidence that increased Ca provision in the form of supplements or dairy foods reduces body weight or body fat in adults
Li et al.* Am J Clin Nutr 2016 (37)	Body weight	MA 33 RCTs and longitudinal studies *N* = 4,733 children and adolescents (9) Postmenopausal women (11) Premenopausal women and men (12)	Ca-rich food, milk, and calcium supplements Mean difference (95% CI) in: Total group -0.01 (-0.02 to 0.0) kg Children and adolescents -0.26 (-0.42 to -0.11) kg Postmenopausal women: -0.14 (-0.54 to 0.26) kg Adult men and premenopausal and older women -0.91 (-1.38 to -0.44) kg	Ca intake in all calcium-intervention groups met the DRI of calcium (1,000 mg/day), whereas calcium intake through diet foods in control groups was below the DRI of calcium	Increased Ca intake through supplements can reduce body weight in participants who have a normal BMI or in children and adolescents, adult men, or premenopausal women No evidence in postmenopausal women
	**Hypertension**				
Cormick et al.* Cochrane Database of Systematic Reviews 2015 (45)	Hypertension, systolic and diastolic blood pressure (BP)	16 RCTs (one of them in children at the age of 11) *N* = 3,048 normotensive people <35 years: 7 trials *n* = 399 >35 years, 9 trials *n* = 2,649	Ca supplementation or food fortification Mean difference (95% CI) Systolic BP -1.43 mmHg (-2.15 to -0.72) Diastolic BP -0.98 mmHg (-1.46 to -0.50)	In age group < 35 years: Systolic BP -2.11 mmHg (-3.58 to -0.64) Diastolic BP-2.61 mmHg (-3.74, -1.49) In age group >35 years Systolic BP -0.96 mmHg (-1.83 to -0.09) Diastolic BP -0.59 mmHg (-1.13 to -0.06)	An increase in Ca intake slightly reduces both systolic and diastolic blood pressure in normotensive people, particularly in young people

* Selection of topics for systematic reviews for the NNR2023 project.

**Table 2:** Characteristics of the studies evaluating calcium intake and cancers

Author	Outcomes	Type of study	Exposure	Exposure	Conclusion
Veettil et al.* JAMA 2021 (57)	**Colorectal cancer**	Umbrella review of 45 meta-analyses describing 109 prospective cohort studies	Dairy products in Schwingshackl et al. 2018 (highly suggestive evidence) Yogurt in Zhang et al. 2019 (convincing evidence) Non-fermented milk in Ralston et a. 2014 (suggestive evidence)	Dietary Ca in Meng et al. 2019 (convincing evidence) Supplemental Ca in Heine-Bröring et al. 2015 (both yes/no and high/low) (suggestive evidence)	Convincing evidence that higher **dietary Ca and yogurt intake is** associated with lower CRC risk
**World Cancer Research Fund** Meat, fish and dairy products and the risk of cancer. 2018 (56)	**Colorectal cancer**	Dose-response meta-analyses in 2017	Dairy products 10 studies (published from 1999 to 2013) Overall risk estimate of 0.87 (0.83 to 0.90) per 400 g dairy products increase per day 9 studies on milk consumption (published from 2001 to 2013) Overall risk estimate of 0.94 (0.92 to 0.96) per 200 g milk increase per day	Dietary Ca 13 studies (published from 1994 to 2010) Overall risk estimate of 0.94 (0.93–0.96) per 200 mg Ca increase per day 7 studies on cheese consumption Overall risk estimated of 0.94 (0.087–1.02) per 50 g cheese per day.	Consumption of dairy products probable decreases risk of CRC
Markozannes et al.* Eur J Cancer 2016 (52)	**Prostate cancer**	An umbrella of evidence	Data were extracted from the 2014 prostate cancer WCRF CUP project, which includes SRs and MAs separately by single foods, nutrients, indices of physical activity, and somatometric factors. The literature search in the CUP was performed in MEDLINE and included RCTs and cohort studies published up to **30th April 2013**	Dietary Ca and non-advanced PCa (7 studies) per 400 mg/day 7% increased risk 1.07 (1.03 to 1.12)	Evidence suggestive for increased risk of non-advanced PCa
**World Cancer Research Fund** Meat, fish and dairy products and the risk of cancer 2018 (13)	**Prostate cancer**	Dose-response MA 2014	Dairy products 15 studies (published from 1999 to 2010) Overall risk estimate of 1.07 (1.02–1.12) per 400 g dairy products increase per day	Diets high in calcium (15 studies) Overall risk estimate of 1.05 (1.02–1.09) per 300 mg Ca increase per day The evidence relating nondairy Ca to prostate cancer was too limited or inconsistent for a conclusion to be made	Limited-suggestive for dairy products: Increases risk of PCa Limited-suggestive for diets high in calcium: Increases risk of PCa
**World Cancer Research Fund** Meat, fish and dairy products and the risk of cancer 2018 (51)	**Breast cancer**	Dose-response MA in 2017	Dairy products and premenopausal breast cancer (7 studies) Overall risk estimate of 0.95 (0.92–0.99) per 200 g dairy products increase per day Diets high in calcium and premenopausal breast cancer (5 studies) Overall risk estimate of 0.87 (0.76–0.99) per 300 mg Ca increase per day	Diets high in calcium and postmenopausal breast cancer (6 studies) Overall risk estimate of 0.96 (0.94–0.99) per 300 mg Ca increase per day	Limited-suggestive for dairy products: Decreases risk of BC (premenopausal) Limited-suggestive for diets high in calcium: Decreases risk of BC (premenopausal) Limited-suggestive for diets high in calcium: Decreases risk of BC (postmenopausal)
Hidayat et al.* Br J Nutr 2016 (50)	**Breast cancer**	MA of prospective studies 9 studies in the dose-response analysis	Ca intake 11 studies published from 2002 and 2013 Overall risk estimate of 0.92 (0.85–0.99) for high versus low intake In the subgroup analysis, the inverse association appeared stronger for premenopausal breast cancer, RR 0.75 (95% CI) 0.59 to 0.96) than for postmenopausal breast cancer 0.94 (0.87 to 1.01)		Dose–response analysis revealed that each 300 mg/day increase in Ca intake was associated with 2% (RR 0·98; 95% CI 0·96, 0·99), 8% (RR 0·92; 95% CI 0·87, 0·98) and 2% (RR 0·98; 95% CI 0·97, 0·99) reduction in the risk of total, premenopausal and postmenopausal breast cancer, respectively

* Selection of topics for systematic reviews for the NNR2023 project.

**Table 3:** Characteristics of the studies evaluating calcium and bone health outcomes

Author Year	Outcomes	Type of study	Exposure	Exposure	Conclusion
Tai et al.* BMJ 2015 (70)	Lumbar spine, total hip, femoral neck, total body, or forearm BMD	SR, MA 59 RCT: 15 dietary sources of calcium (*N* = 1,533) 51 calcium supplements (*N* = 12,257) Older adults (>50 years)	Dietary sources of Ca or Ca supplements (with or without vitamin D) Ca/milk supplement Study duration of 12 to 48 months Mean change (95% CI) in MA Dietary Ca: LS 0.7 (0.3 to 1.2) Femoral neck 1.8 (1.1 to 2.6) Total hip 1.5 (0.7 to 2.4) Forearm 0.1 (-0.3 to 0.4)	Mean change (95% CI) in MA Supplements: LS 1.1 (0.7 to 1.6) Femoral neck 1.0 (0.5 to 1.4) Total hip 1.4 (0.6 to 2.3) Forearm 0.7 (0.4 to 1.1)	Increases in BMD were similar in trials of dietary and supplemented Ca BMD increased by 0.6 to 1.8% Increases were similar with Ca monotherapy versus CaD, with Ca doses of ≥1,000 versus 500 mg/day, and when the baseline dietary Ca intake was <800 versus ≥800 mg/day
Silk et al.* Int J Sport Nutr Exerc Metab 2015 (69)	Femoral neck, Lumbar spine, Total body, and Total hip BMD	SR and MA 11 studies 6 in MA men 16–84 years, mean age 55 ≥6 months intervention *N* = 867	Ca with or without vitamin D supplementation on BMD in males Effect size, ES (95% CI): Total body 0.644 (0.406 to 0.883), Total hip 0.483; (0.255 to 0.711) Femoral neck 0.402 (0.233 to 0.570) Lumbar spine 0.306 (0.173 to 0.440)		Limited evidence appears to support the use of Ca and CaD supplementation for improving BMD in men
**Handel et al**.* Am J Clin Nutr 2015 (6)	Childhood fx	SR of 18 observational studies (2 longitudinal, 16 cross-sectional) MA: 9 studies Boys and girls aged 2 to 13 years	Dietary Ca intake or serum nutritional concentrations		Milk avoidance, high cheese intake, high energy or sugar-sweetened beverage intakes were associated with an increased fx risk
van den Heuvel & Steijns* Nutr Res Rev 2018 (68)	BMC or BMD and fx risk in children and adults	Review of 4 SRs, 2 MA in children 2 SRs, 4 MA in adults	Dairy products, as reported in SRs and MAs on RCTs in the case of bone mineralization or prospective studies in the case of fx risk Total body BMC of girls improved by 45–50 g over 1 year when the daily baseline Ca intake was lower than 750 mg	In women, increasing Ca intake from dairy sources with or without vitamin D increased BMD by 0·7 to 1·8% at the lumbar spine, total body, total hip, or femoral neck at 2 years	Dairy product improved BMC in girls No conclusion can be drawn to childhood fx risk Based on 4 SRs/meta-analyses and 2 prospective studies with contrasting results, dairy products, either or not fortified with vitamin D, do not significantly reduce hip fx risk
Bolland et al.* BMJ 2015 (71)	Total, hip, vertebral, and forearm fx	SR, MA 2 RCT of dietary Ca 50 reports of 44 cohort studies 26 RCTs of Ca supplements Older adults (>50 years) *N* = 58,573	Dietary Ca (*n* = 37), milk (*n* = 14), or dairy intake (*n* = 8) Ca supplement Dietary Ca: No relation with total, hip, vertebral, or forearm fx 74% of the studies reported neutral associations between dietary calcium intake and fracture outcomes	Ca supplements RR (95% CI) Total fx 0.89 (0.81 to 0.96) Vertebral fx 0.86 (0.74 to 1.00) Hip fx 0.95 (0.76 to 1.18) Forearm fx 0.96, (0.85 to 1.09)	No evidence that increased dietary calcium intake prevents fractures Ca supplements have small inconsistent benefits on fracture prevention. Mean decline in total fx and vertebral fx was 11% and 14%, respectively. No benefit in hip or forearm fx
Zhao et al.* JAMA 2017 (74)	Incidence of hip, vertebral, non-vertebral and total fx	SR and MA of 33 RCTs *n* = 51,145 Adults > 50 years *N* = 51,145	Ca, vitamin D, or combined CaD supplements RR (95% CI) Ca: Hip fx 1.53 (0.97 to 2.42) Vertebral fx 0.83 (0.66–1.05) Non-vertebral fx 0.95 (0.82–1.11) Total fx 0.88 (0.75–1.03)	RR (95% CI) CaD: Hip fx 1.09 (0.85 to 1.39) Vertebral fx 0.63 (0.29–1.40) Non-vertebral fx 0.88 (0.75–1.03) Total fx 0.90 (0.78–1.04)	No significant associations were found between Ca, vitamin D, or combined CaD supplements and the incidence of non-vertebral, vertebral, or total fx The results were generally consistent regardless of the dose of Ca or vitamin D, sex, fracture history, Ca intake, and baseline serum 25-(OH)D concentration
**Kahwati et al.** JAMA 2018 (73)	Incident fx, mortality, kidney stones, cardiovascular events, and cancer	SR 11 RCTs or observational studies Adults 50 years or older *N* = 51,419	Supplementation of vitamin D, Ca, or combined CaD: ARD (95% CI) Total fx, -0.35% (-1.02 to 0.31%) Hip fx, -0.14% (-0.34 to 0.07%) Ca alone: ARD Kidney stones, 0.0 (-0.87 to 0.87%), CaD: an increased ARD, 0.33 (0.06 to 0.60%)	Vitamin D decreased total fx incidence ARD -2.26% (-4.53 to 0.00) but not hip fx ARD -0.01% (-0.80 to 0.78) CaD was not associated with an increase in cancer incidence ARD -1.48% (-3.32 to 0.35)	CaD supplementation had no effect on total fracture or hip fracture incidence, or on all-cause mortality or CVD CaD supplementation was associated with an increased incidence of kidney stones, but Ca alone was not associated with an increased risk Ca alone or CaD supplementation was not associated with an increase in cancer incidence
**Yao et al.** JAMA 2019 (75)	Fractures Any or the hip	SR and MA in observational studies and RCTs *N* = 39,141 participants *N* = 6,278 fx, *N* = 2,367 hip fx	Supplementation with vitamin D alone or in combination with Ca MA of 6 RCTs of combined supplementation with vitamin D and Ca was associated with RR (95% CI) for Any fx 0.94 (0.89–0.99) Hip fx 0.84 (0.72–0.97)	Vitamin D alone (daily or intermittent dose of 400–30,000 IU, yielding a median difference in 25(OH)D concentration of 8.4 ng/mL) did not reduce the risk RR for All fx 1.06 (0.98–1.14) Hip fx 1.14 (0.98–1.32)	CaD (daily doses of 1,000–1,200 mg and 400–800 IU) found a 6% reduced risk of any fx and a 16% reduced risk of hip fx
**Tricco et al.** JAMA 2017 (76)	Injurious falls	Network meta-analysis 54 RCTs *N* = 41,596 Age ≥ 65 years	Exercise, vision assessment and treatment, environmental assessment and modification, multifactorial assessment and treatment, Ca, and vitamin D supplementation OR (95% CI) Exercise 0.51 (0.33 to 0.79)	Exercise plus vision assessment and treatment 0.17 (0.07 to 0.38] Plus environmental assessment and modification 0.30 (0.13 to 0.70) Plus multifactorial assessment and treatment (e.g. comprehensive geriatric assessment), Ca supplementation, and vitamin D supplementation (OR, 0.12 [0.03 to 0.55])	Exercise alone and various combinations of interventions were associated with lower risk of injurious falls compared with usual care
**Iuliano et al.** BMJ 2021 (77)	Fx, falls, all cause mortality	Cluster RCT *N* = 7,195 permanent residents (4,920 (68%) female) Mean age 86.0 years in 60 facilities (30+30)	Dairy products versus usual menus	HR (95% CI) for All fx 0.67 (0.48 to 0.93) Hip fx 0.54 (0.35 to 0.83) Falls 0.89 (0.78 to 0.98) Mortality 1.01 (0.43 to 3.08)	Improving Ca and protein intakes by using dairy foods reduced 11% the risk of falls and 33% the risk of all fractures in aged care residents There was no difference in mortality

* Selection of topics for systematic reviews for the NNR2023 project.

**Table 4:** Characteristics of the studies evaluating calcium and pregnancy related health outcomes.

Author	Outcome	Type of study	Exposure	Exposure	Conclusion
An et al.* Int J of Nursing Practice, 2015 (79)	Hypertensive disorders of pregnancy and related problems	MA of multicenter RCTs Four studies included from 1991 to 2006	Ca supplementation 16% reduced risk of gestational hypertension RR, 0.91 (0.84 to 0.99)	No association with pre-eclampsia	Ca supplementation appears to reduce the risk of hypertension in pregnancy.
Patrelli et al.* J Matern Fetal Neonatal Med 2012 (81)	Preeclampsia	MA 16 RCT studies included from 1987 to 2012	Ca supplementation in pregnant women with low Ca intake (seven studies, 10 154 patients) demonstrated a significant reduction in the incidence of preeclampsia (RR=0.73; 95% CI = 0.61–0.87)	A statistically insignificant relationship (p = 0.09) between Ca supplementation during pregnancy and the risk of preeclampsia (RR = 0.88; 95% CI=0.77–1.02) in patients with adequate Ca intake	Additional intake of Ca during pregnancy is an effective measure to reduce the incidence of preeclampsia, especially in populations at high risk of preeclampsia due to ethnicity, age, high BMI and in those with low baseline Ca intake
Hofmeyr et al.* Cochrane Database of Syst Rev 2014 (80)	Pregnancy and related problems	Cochrane review 13 RCTs N= 15,730 women	High-dose (≥1 g /d) or low-dose Ca supplementation vs. placebo or no Ca RR (95% CI) Pre-eclampsia 0.45 (0.31 to 0.65) Preterm birth 0.76 (0.60 to 0.97) Risk of high blood pressure (BP) 0.65 (0.53 to 0.81)	Updated 2017 and 2019, but only one additional trial was found in each year.	Ca supplementation (≥1 g) halves the risk of preeclampsia in women with low Ca intake. 35% reduction in the risk of gestational hypertension, with the greatest effect in women at high risk and those with low Ca diets.
Buppasiri et al. * Cochrane Database Syst Rev 2015 (82)	Maternal, fetal, and neonatal outcomes (not hypertension)	Cochrane review 23 RCTs N=18,578 pregnant women	Ca supplements RR (95% CI) Preterm births less than 37 weeks’ gestation 0.86 (0.70 to 1.05) less than 34 weeks’ gestation 1.04 (0.80 to 1.36) Infant low birthweight 0.93 (0.81 to 1.07) Compared to the control group, women in the Ca supplementation group gave birth to slightly heavier birthweight infants Mean difference (95% CI) 56.40 (13.55 to 99.25)	Maternal and neonatal BMD No evidence to support the benefit of Ca supplementation in increasing BMD in pregnant women In infants, statistically significant difference between treatment and placebo or no treatment in total body and tibial BMD.	No clear benefits in prevention of preterm birth or low infant birthweight. A small probably clinically insignificant difference of 56 g in mean infant birthweight may be. Few short-term benefits other than slight increases fetal birthweight and neonatal bone mineral density. Data is limited to assess long-term benefits of Ca supplementation, such as osteoporosis in later life.

* Selection of topics for systematic reviews for the NNR2022 project

SR= systematic review; MA= meta-analysis; RCT= randomized controlled trial; BMD= bone mineral density, CA= Calcium; CaD= calcium supplement with vitamin D; RR= Relative risk/Risk ratio

The evidence gathered was mainly related to following topics: pregnancy health (hypertensive disorders, pre-eclampsia, and fetal/neonatal outcomes), skeletal health (bone mineral density [BMD], fractures, and osteoporosis), cardiovascular health, cancers, with the main focus on colorectal, breast, and prostate, and body weight and obesity, with the purpose of updating the qSR produced for the 5th edition of the Nordic Nutrition Recommendations (14), which showed only minor or inconsistent benefits of calcium supplementation on health outcomes.

## Physiology of calcium metabolism and bone growth

Calcium is the most abundant mineral in the body. At full-term birth, the human infant has accrued about 26 to 30 g of calcium, most of this in the skeleton as calcium hydroxyapatite (Ca_10_[PO_4_]_6_[OH]_2_), while the adult human body contains about 1,200–1,400 g of calcium. Over 99% of calcium is stored in bones and teeth providing structure and strength for the skeleton to function mechanically. Less than 1% of total body calcium is found in soft tissues and body fluids, where it serves as an essential regulator of several body functions, such as muscle contraction, function of the nervous system, and blood clotting. Calcium is present in blood in three different forms: as free Ca^2+^ ions, bound to protein (about 45%), and complexed to citrate, phosphate, sulfate, and carbonate (about 10%) (15).

Calcium is found naturally in some foods, added to others, available as a dietary supplement, and present in some medicines (such as antacids). Serum calcium is tightly regulated and does not fluctuate with changes in dietary intakes, due to its vital importance. Only free ionized calcium is of physiological importance, and its concentration is approximately half of the total concentration (16). It has not been established whether calcium consumption of less than 2,500 mg/day contributes to arterial calcification and cardiovascular diseases (CVD) in the general adult population. It has also been speculated that calcium loading from supplements, that is bolus consumption of large amounts in one dose, may be more likely to accelerate arterial calcification than smaller doses from foods over a day, especially in older adults (17–19).

**Fig. 2:**
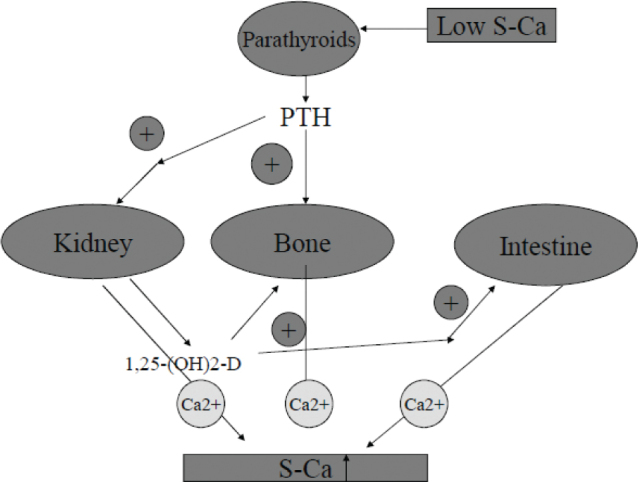
Endocrine feedback system that maintains serum calcium levels: Involvement of 1,25(OH)_2_D and parathyroid hormone (PTH) (from Nordic Nutrition Recommendations 2012, reference 33).

The requirement for calcium is currently solely based on the size of the calcium reserve, that is on total skeletal and regional bone mass. Bone mineral as a reservoir of calcium helps to maintain a constant concentration of blood calcium. Activated vitamin D (1,25-(OH)_2_D) contributes to the maintenance of serum calcium levels by increasing the absorption of calcium in the upper small bowel and by stimulating osteoclastic bone resorption. Bone itself undergoes continuous (re)modeling, with constant resorption and deposition of calcium into new bone. The rapid release of mineral from the bone is essential to maintain adequate levels of ionized calcium in serum (2, 20, 21) (Fig. 2).

In general, modeling refers to alterations in the shape of bone, whereas remodeling refers to the lifelong renewal process of the skeleton. Bone is constantly renewed at an average rate of 8–10% per year, and the body’s need for calcium relative to skeletal growth and remodeling varies by life stage. Anatomically, there are two types of bone tissues: cortical (compact) and trabecular (cancellous) bone. Cortical bone constitutes approximately 80% of the skeletal mass and trabecular bone 20%.

Three cell types are typically associated with bone homeostasis: osteoblasts (bone-forming cells), osteocytes (mature bone cells), and osteoclasts (bone-resorptive cells). Trabecular bone has an active metabolic role, while the main function of cortical bone is to provide structure and protection (22). Bone turnover markers are widely used in clinical trials for measuring bone formation or resorption, but these markers are not useable in estimating calcium (in)sufficiency (23).

Calcium must be ingested with the diet in sufficient amounts to allow for calcium deposition during bone growth and modeling and to compensate for obligatory intestinal, fecal, and dermal losses during the lifetime. The instant absorbability of calcium is affected by the amount and source of calcium. Calcium carbonate is highly insoluble unless converted into calcium chloride by hydrochloric acid (HCl), but other organic salts such as calcium citrate, calcium lactate, and calcium gluconate are more soluble and ionizable at neutral pH (24). The fractional absorption of calcium is lower with higher calcium load, and foods vary widely in calcium content. Calcium leaves the body mainly not only in urine and feces but also in other body tissues and fluids, such as sweat. High intakes of sodium increase urinary calcium excretion, and this may affect bone calcium balance. A high intake of sodium appears to have a detrimental effect on bone calcium balance when the intake of calcium is low (25). In contrast, adding more potassium to a high-sodium diet might help decrease calcium excretion, particularly in postmenopausal women (26).

Infancy through late adolescence is characterized by positive calcium balance. In female adolescents and adults, even within the normal menstrual cycle, there are measurable fluctuations in calcium balance owing to the effects of fluctuating sex steroid levels and other factors on the basal rates of bone formation and resorption. Later in life, menopause and age-related bone loss lead to a net loss of calcium due to enhanced bone resorption.

Plant-based diets can lead to lower intakes of nutrients, such as calcium, vitamin D, and some B-vitamins (27). As well, different protein sources (plant and animal) with varying amino acid profiles may have diverse effects on BMD and bone turnover. Recent studies have suggested that vegans have higher levels of circulating bone turnover markers compared to omnivores, which may, in the long-term, lead to poorer bone health (28–31). Recent studies have suggested that vegan diet may increase bone metabolism. In a 12-week trial, Itkonen et al. showed that partial replacement of animal-based protein with plant-based protein sources increased bone turnover and mineral metabolism, indicating a possible risk for bone health deterioration in healthy adults. This result was probably caused by lower calcium and vitamin D intakes from diets containing more plant-based proteins, and it is unclear whether differences in protein intake or quality play a major role. The plant-based protein sources were soy-based tofu, cereal products, fava beans, peas, and seeds and nuts (32). However, more prospective research is needed to clear the impact of plant-based diets on bone health.

## Assessment of nutrient status

Because there are no direct biomarkers of calcium status, the quantity of sufficient dietary calcium is based on bone growth and turnover. Moreover, the interplay between the dynamics of calcium and vitamin D often complicates the interpretation of data relative to calcium requirements, deficiency states, and excess intake.

## Dietary intake in Nordic and Baltic countries

According to a paper by Lemming and Pitsi (34), the Nordic and Baltic countries share both similarities and differences in food culture, which, in turn, reflect differences in average food consumption and nutrient intakes across countries. Relating to calcium intake, the consumption of dairy product ranges between the lowest of 124 g in Lithuanian women to the highest of 480 g in Finnish men. Available data for the Nordic countries, using either 24-h recall or food records, show an average daily calcium intake ranging from 811 mg among women in Norway to 1,188 mg among men in Denmark. However, mean calcium intake remains below the recommendations in the Baltic countries, with the average calcium intake ranging from 546 mg among women in Lithuania to 768 among men in Estonia and Latvia. The mean intake of calcium among children and teenagers in Lithuania is below the recommended intake and among girls in the age of 10 to 17 years in Estonia. Available data from Norway and Sweden show that the average calcium intake is close to or above the NNR2012-recommended intake (900 mg) for children and teenagers (33), although data from Norway show lower calcium intake on average compared to Sweden (34).

Looking at dairy and dairy product intake, the food grouping differed between the countries; for example, in Finland, the dairy product group included cheese, whereas Sweden did not include cheese in dairy products (34). These differences will have an effect on the estimated total calcium intake from dairy products when compared between countries.

Most of the dietary calcium comes from dairy products, but some non-dairy products can also contribute notably, for example vegetables, starchy foods, dried fruits, and water. Calcium-fortified foods are also widely used and can help people to fill calcium gaps in their daily diets (35).

## Health outcomes relevant for Nordic and Baltic countries

### Obesity and body weight

Obesity often coexists with low calcium intake and vitamin D insufficiency, but evidence for an association between calcium intake and body weight is contradictory. According to one hypothesis, increasing calcium intake during weight loss should result in greater fat loss and reduced loss in fat-free mass.

Li et al. analyzed weight changes from 33 studies, 9 enrolled as children, and there was a negative correlation between calcium supplementation and weight changes in children, the mean (95% CI) being -0.26 kg (-0.41 to -0.11) favoring the experimental group. For adult body weight, despite a wide range of calcium intakes (from supplements or from dairy and non-dairy dietary sources), evidence did not support that increased calcium intake would accelerate weight or fat loss in obesity (36, 37) (Table 1).

### Cardiovascular outcomes

Calcium supplements are mostly recommended and used to prevent fractures in postmenopausal women (38–40). This use was discouraged due to raised concerns about a possible increase in CVD risk associated with calcium supplementation (4). However, so far, results have remained contradictory, and much criticism has been presented against this suggested risk, since to date, no evidence has been presented from trials with CVD as the primary endpoint.

Lewis et al. undertook a meta-analysis of randomized controlled trials (RCTs) with placebo or no-treatment control groups to determine if calcium supplements with or without vitamin D increase all-cause mortality and coronary heart disease (CHD) risk in elderly women (41). They included both published and unpublished data, and heart disease and its common clinical manifestations were verified by clinical review, hospital record, or death certificate. There was no increase in the relative risk of cardiovascular events or in all-cause mortality (41).

On the contrary, Myung et al. found that calcium supplements increased the risk of CVD by about 15% in healthy postmenopausal women (42) (Table 1).

Chung et al. did not find statistically significant differences in risk for CVD events or mortality between groups with or without dietary or calcium supplements. Also, no significant associations were found relating to calcium intake (≥1,000 vs. <1,000 mg/day). They concluded that calcium intake within tolerable upper intake levels (2,000 to 2,500 mg/day) was not associated with CVD risk in generally healthy adults (43). Interestingly, Asemi et al. found a significant relationship between the total calcium intake and an increased risk of CVD mortality in studies with a long follow-up time of >10 years, and a significant protective association in studies with a mean follow-up of <10 years (44) (Table 1).

Inverse relationship between calcium or dairy intake and BP has been reported in various epidemiological studies, typically showing that reduced intake of calcium is associated with higher BP and increased risk of developing hypertension. Increased calcium intake slightly reduced BP, and mean decline (95% CI) in systolic BP was 1.43 mmHg (0.72 to 2.15), and that of in diastolic BP was 0.98 mmHg (0.50 to 1.46) in normotensive people. The effect was consistent in both sexes at ages from 11 to 82 years old, but the reduction was greater among younger people. The effect was higher with doses of calcium above 1,000 mg/day. None of the studies reported adverse events. One of the 16 trials was conducted in children 11 years of aged, and one in teenagers (45) (Table 1).

Although the report of Bolland et al. in 2008 (4) is of concern, much criticism has been presented against these findings due to several relevant limitations; for example, none of the studies had been powered to significantly detect cardiac events, cardiovascular events were not a primary outcome, the events may not have been well adjudicated, and the methodology did not allow the results to be generalizable to a broader population (45). Moreover, most of the studies did not report total calcium intake, but instead supplemented calcium with 1,000 to 1,200 mg of calcium per day. The events may therefore be associated with calcium intakes that consistently exceed the recommended daily intake of older adults. Also, one of the largest prospective cohort studies, the UK Biobank study, with over 500,000 middle-aged men and women, found no association between the use of calcium supplements and risk of hospital admission or death after ischemic cardiovascular events. The findings were similar in men and women, and neither calcium with vitamin D supplement changed the results (46).

### Cancers

According to the World Health Organization (WHO) in 2019, cancer is the first or second leading cause of premature death (before the age of 70 years) in majority of countries in the world (47). The most common cancers worldwide in 2020 were diagnosed in breast (12%), lung (11%), colorectal (10%), prostate (7%), and stomach (6%) (48). In the present review, the emphasis is on the most common cancers, such as breast, colorectal, and prostate. The continuous update project (CUP) conducted by the WCRF has summarized the evidence between calcium intake and risk of the above-mentioned cancer types (13). When judging the evidence in CUP, only strong evidence is used for the basis of recommendations to lower cancer risk.

Studies have shown that calcium signaling pathways in cells can influence various processes important in cancer progression such as proliferation, invasion, and cell death (49). The CUP reported in 2017 that the evidence is limited-suggestive between high intake of dairy products and diets high in calcium and lower risk of premenopausal breast cancer. The evidence for diets high in calcium and lower risk of postmenopausal breast cancer was also categorized as limited-suggestive (13) (Table 2).

One other meta-analysis, published in 2016, has reported similar results for breast cancer as WCRF (50). One of the meta-analyses showed that the inverse association was weaker for calcium supplements alone compared with calcium from diet (50).

Prostate cancer usually develops slowly and is more often localized when diagnosed, whereas advanced disease is less common. The WCRF reported in 2014 that the evidence was limited between high dietary calcium intake and total prostate cancer risk. Furthermore, dose-response meta-analysis examining calcium supplements showed no association with the risk of total prostate cancer, but a significant positive association for fatal prostate cancer (51) (Table 2).

In 2016, an umbrella review was performed on the evidence identified in the WCRF on diet, body size, physical activity and risk of prostate cancer to further evaluate the strength of the studies and to consider the extent of potential biases. This umbrella review came to the same conclusion regarding high calcium intake and risk of prostate cancer (52).

Various mechanisms could explain the positive association found in some, but not all studies, between dairy consumption rich in calcium and prostate cancer (Table 2). Dairy consumption increases blood levels of insulin-like growth factor-1 (IGF-1), which is suggested to be a risk factor for prostate cancer (53). Moreover, calcium might act through calcium-sensing receptor in prostate cells (*CaSR*) since genetic variations across *CaSR* have been shown to be associated with fatal prostate cancer (54). It has been suggested that calcium intake might affect early prostate cancer development (≥12 years before diagnosis) but only at very high levels of intake (>2,000 mg/day) (55). Finally, dairy products are also rich in phosphorus, which has been reported to be an independent risk factor for fatal- and high-grade prostate cancer (55).

Colorectal cancer seems to be affected by numerous lifestyle factors. In the CUP analysis from 2017, the results were quite consistent for dairy products, milk, cheese, and dietary calcium, where higher consumption was associated with lower risk of colorectal cancer. The CUP panel concluded that the consumption of dairy products probably protects against colorectal cancer, and the evidence was categorized as convincing (56). In an umbrella review published in 2021, it was concluded that the evidence was convincing for higher intakes of dietary calcium and yogurt and lower risk of colorectal cancer, while the evidence for dairy products was considered highly suggestive and calcium supplements, and non-fermented milk was considered suggestive (57) (Table 2).

Possible mechanisms include many plausible pathways, such as through calcium signaling (49), and additionally casein and lactose in milk might increase calcium bioavailability (58). In addition, calcium binds to bile acids and free fatty acids in the gut reducing their contact to the colonic epithelial cells. Furthermore, lactic acid-producing bacteria might also protect against colorectal cancer (59).

In a recent study from UK on attributable risk factors related to lifestyle, it was calculated that 7% of colorectal cancer cases could be prevented if the consumption of dietary calcium were above 700 mg/day (60).

Although the evidence was convincing, the CUP panel did not give recommendation on dairy consumption for this matter because of the suggestive evidence related to increased risk of prostate cancer.

Two other SRs were identified in the PubMed/MEDLINE search. One showed no association between the dietary calcium intake and lung cancer risk (61). The other SR was on ovarian cancer, where a protective association was observed, although the authors stated that larger cohort studies were needed on this subject (62).

### Bone health

Dietary calcium intake and skeletal calcium requirements vary widely across the various stages of the life. Fractional calcium absorption is highest (about 60%) in breastfed infants, declines in the infant transition into childhood, only to rise again in early puberty, when modeling of the skeleton is maximal, and then decreases again to 15 to 20% in young adults, and thereafter declines gradually. Calcium absorption is increased in pregnant and lactating women compared to non-pregnant women (63–66).

The critical time for optimal calcium intake occurs during the formative bone growth years, and high calcium intake in later life does not seem to promote an increase in BMD and thereby reduce bone fracture risk in adults. With respect to the effect of optimal calcium intake, the window of opportunity to build strong bones closes by approximately the beginning of the third decade of life. Since the calcium reserve is vast relative to the cellular and extracellular metabolic pools of calcium, dietary insufficiency virtually never impairs biochemical functions that are dependent on calcium. However, since bone strength is a function of bone mass, any decrease in bone mass (calcium reserve) will produce a corresponding decrease in bone strength (19, 63).

Previously, it has been shown that calcium supplementation through the diet or through supplements modestly but significantly increases total body and lumbar spine bone mineral content (BMC) in children (67). Recent SRs confirmed these results. Plain dairy products or those fortified with calcium and/or vitamin D improved total body BMC by 50 g (95% CI 24 to 77 g) over 1 year. The effect was most profound when the daily baseline calcium intake was lower than 750 mg. On the other hand, as a threshold nutrient, increasing calcium intake would only be expected to benefit bone health if calcium supply was a limiting factor impacting on either the density or architecture of bone. The role of dairy products was less clear for regional bone sites. Baseline calcium intake seemed to explain most of the observed statistical heterogeneity (68) (Table 3).

SRs and meta-analyses relating to bone mineralization, osteoporosis, or fracture risk have been published regularly with a growing number of studies, yet young women have not been in the center of attention, and we did find neither any SRs nor even RCTs executed in this millennium examining the effects of calcium intake on premenopausal women’s bone health.

As well, the effects of calcium intake on adult male bone health have not inspired research, and the number of studies to adequately determine the efficacy of calcium with or without vitamin D is low. Based on one meta-analysis, in which mean differences in BMD between the groups were presented in Hedge’s g – a measure of effect size (ES), the ES (95% CI) for the total hip and femoral neck, which are the most important bone sites for osteoporotic fractures, was 0.483 (0.255 to 0.711) and 0.402 (0.233 to 0.570), respectively (69) (Table 3). Thus, limited evidence appeared to support the use of calcium and vitamin D supplementation for improving BMD in older males. The estimation of fracture risk reduction is not possible, and results of young males are based on only one study.

Most RCTs evaluating effects of calcium, or calcium with vitamin D, on BMD or the rate of fractures among older adults are in postmenopausal women. Tai et al. conducted an SR and meta-analysis in adults above 50 years of age and identified 59 eligible RCTs either with dietary sources of calcium or calcium supplements. This is the largest recently published SR, but only four of those studies were in men, and three included both sexes. Increased dietary or supplemental calcium intake produced small non-progressive 1–2% increase at lumbar spine and hip BMD with little further effect after a year. Dairy products did not beat the effects of calcium supplements; increases were similar in trials with dietary calcium and calcium supplements, and when using calcium alone or with vitamin D. As well, the changes were independent of calcium doses (calcium ≥1,000 vs. 500 mg/day) or baseline dietary calcium intake (over or below 800 mg/day) (70). These increases are small and unlikely to lead clinically meaningful reductions in fractures (Table 3).

### Fractures

Data from RCTs about dietary exposures and later fracture rates are limited. However, Handel et al. carried out an SR and meta-analysis based on case–control studies that examined the association between the dairy calcium intake and childhood fractures (6) (Table 3). Although milk avoidance and low calcium intake seemed to be associated with an increased fracture risk, the association was not consistent, and in the pooled meta-analysis, no significant differences in calcium intake were found in the prevalence of fractures between the case and control groups.

It is also challenging to estimate dietary intake accurately in children, which may cause bias in the estimated nutrient intake. It must also be considered that children who avoid milk prefer noncarbonated or carbonated high energy, sugar-sweetened beverages over water or calcium-fortified beverages. A high proportion of dairy products may also cause unbalanced diet, which complicates the analysis (6). A one-time assessment of dairy product intake may not accurately predict the intake over a long follow-up; hence, predicting fracture risk based on childhood or adolescence dietary behavior can be rather difficult, if not impossible (68).

In adults, Bolland et al. undertook an SR of studies of dietary calcium or calcium supplements in adults over 50 years of age with fractures as an endpoint. Relationships between dietary calcium and fractures were based on cohort studies, while relationships between calcium supplements and fracture risk were based on RCTs. For milk and dairy intake, most analyses (≥75%) found no associations with fractures, or associations were weak (71).

The association between calcium supplements and fragility fractures is more commonly evaluated than dietary calcium sources. In a meta-analysis of 20 RCTs, calcium supplements reduced the relative risk (95% CI) of total fractures by 11% (4 to 19%) and vertebral fractures by 14% (0 to 26%) (12 RCTs), but no effect on forearm or hip fractures was found. However, results were not consistent. Frail older women living in residential care (one trial) with low dietary calcium intake and low vitamin D concentrations showed a significant reduction in the hip fracture risk when supplemented with calcium combined with vitamin D (72). More recent meta-analyses have been consistent with these findings (73, 74). There were no significant differences within subgroups based on the dose of calcium or vitamin D, sex, fracture history, dietary calcium intake, and baseline serum 25-hydroxyvitamin D concentration (74). Also, the combination of calcium with vitamin D did not turn out to be more beneficial in community-dwelling older adults (Table 3).

In contrast, Yao et al. demonstrated a marginally significant reduction of 6% (1 to 11%) in the risk of any fracture and 16% (3 to 28%) in the risk of hip fracture with combined calcium and vitamin D (75). While benefits of increased calcium were even at its best rather modest, a network meta-analysis of Tricco et al. showed that exercise alone may prevent half of the injurious falls compared with the usual care, odds ratio (95% CI) being 0.51 (0.33 to 0.79), and various combinations of interventions were associated with even greater decline (76) (Table 3).

Dietary calcium intake was not associated with fracture risk, and evidence that calcium supplements could be more effective in fracture prevention was weak and inconsistent. These findings do not support the routine use of calcium supplements with or without vitamin D for fracture prevention in community-dwelling older people. However, calcium with vitamin D may be effective for older adults in institutional care. A recent large 2-year cluster randomized controlled trial showed that supplementation using high calcium, high protein dairy foods reduced falls and fractures in vitamin D replete older adults in aged care (77) (Table 3).

Although calcium supplementation, whether with pills, fortified foods, or dairy, consistently increases skeletal mass gain and bone density in children and adolescents from 1 to 5% (2), and 1 to 2% in adults (68, 70), fall-related osteoporotic fractures are an increasing problem of aging populations. Prevalence of falls among individuals aged 65 years or older in the US was 36% in 2010 (78). The persistence of low calcium intake, even in those participants included in the treatment-arm of the RCTs due to low or sub-optimal treatment adherence, is an important issue, since treatments cannot work if they are not taken. This problem may be less important in studies performed in institutions since the administration of supplements may be more controlled (70, 72). However, the most effective single mean to decline a risk of fractures is falls prevention, and exercise alone may prevent even half of the injurious falls (76).

### Pregnancy-related outcomes

In settings where dietary calcium is low, supplementation is an important strategy to reduce the serious consequences of pre-eclampsia. Where high-dose supplementation is not feasible, the option of lower dose supplements (500 to 600 mg daily) might be considered in preference to no supplementation. Calcium supplementation during pregnancy was associated with a reduction in risk of gestational hypertension, pre-eclampsia, neonatal mortality, and preterm birth mainly in developing countries (79–81) (Table 4).

An SR by Buppasiri et al. evaluated effects of calcium supplementation on maternal, fetal, and neonatal outcomes (other than preventing or treating hypertension), and possible side effects. Calcium-supplemented women gave birth to slightly heavier infants mean difference (95% CI) in birthweight being 56.40 g (13.55 to 99.25 g). Calcium supplementation did not reduce preterm birth, low infant birthweight, or had any effect on maternal weight gain. Dosage, prescription timing, and the type of calcium supplementation did not affect these outcomes.

However, the heterogeneity among the studies was high (82) (Table 4). Although mean calcium intake is the same as the recommended intake in the Nordic countries, calcium intake may not be sufficient in subgroup with low intake of dairy products and/or fortified products.

The evidence identified in the 2021 literature search mainly confirmed earlier findings. Calcium intake/supplementation during pregnancy may protect against the risk of developing hypertension and pre-eclampsia, especially in low-income countries, but no benefits were found for neonatal health or prevention of preterm birth. Although these results may not be easily generalizable in the Nordic countries, it is important to take into consideration that ethnic diversity is widening in the Nordic countries, and the number of young women with low calcium intake may increase.

### Total mortality

In an SR from 2015, 22 prospective studies were included in the meta-analysis to assess the association among total, dietary, and supplemental calcium intake, and mortality from all-causes, CVD, and cancer. An increased risk of mortality due to CVD was observed for higher total calcium intake in studies where mean follow-up was 10 years or longer, while protective association was observed for all-cause mortality and CVD mortality among studies with a mean follow-up of less than 10 years. Moreover, supplemental calcium intake was inversely associated with all-cause mortality (44) (Table 1).

### Adverse events

Most studies agree that adequate calcium intake is important for bone health and several major physiologic functions. Although the maintenance of bone health continues to be an important goal of adequate dietary calcium consumption, excessive use of calcium supplements increases the risk of harms, including kidney stones, hypercalcemia, and minor side-effects such as constipation, or even hospitalization with acute gastrointestinal symptoms. Even healthy kidneys have limited capability of eliminating excessive calcium in the diet (18). Overall, the data indicate that the calcium content of foods does not cause stone formation but may be protective against it, while supplemental calcium is associated with an increased risk for kidney stones. As an explanation has been suggested that supplemental calcium, as it is taken more in bolus form than dietary calcium, causes an increase in urinary calcium and, thus, has higher propensity to cause stone (83).

## Requirements and recommended intakes

Worldwide, the best estimate of average dietary calcium intake among adult population ranges from incredibly low 175 mg/day in Nepal to over 1,200 mg/day in Iceland. Generally, in Asian and African countries and in South America, mean calcium intake may be 500 mg/day or less, while in North-European countries the mean intake is around 1000 mg/day or more (84). Several authorities (e.g. WHO, the US Institute of Medicine [IOM], European Food Safety Authority [EFSA], and NNR) have set recommendations for calcium intake, and Western recommendations for calcium intake for adults range from 700 mg (EFSA) to 1,200 mg (IOM).

The same balance data from the studies, which were used to derive recommendations for North American adults (IOM), were further analyzed by EFSA, with some important differences. First, data from additional studies in which calcium supplements were given (not included in the analysis of the balance by Hunt and Johnson [85]) were added to the database. Second, individual data from adults <25 years were excluded, as there is evidence that additional calcium continues to be deposited in bones after they have ceased growing.

In NNR2012, the recommended intake of 800 mg/day from NNR2004 was maintained for adults over 20 years of age, as no strong evidence has emerged to justify a change (33). The recommended intake for adolescents of 900 mg/day was extended to young adults, noting that some bone mass is still accreted beyond 17 years of age, and that the increased demand for calcium is also reflected in a higher absorption efficiency up to the age of 24 years.

Although most guidelines acknowledge the increased demand of calcium during pregnancy, the fetal need for calcium is met by maternal physiological changes, primarily through increased calcium absorption. The recommendation for pregnant and lactating women is the same as for non-pregnant and non-lactating women. Only in adolescents, whose skeleton is still growing, pregnancy could theoretically reduce peak bone mass and increase the long-term risk of osteoporosis (3, 86).

The consumption of adequate dietary calcium can be accomplished within a variety of dietary preferences, although dairy products are generally the most important food source in European countries.

In European diets, about 45 to 70% of the dietary calcium intake is provided by dairy products. Some people avoid all dairy because of allergies or personal choice. Then, the consumption of dark green vegetables and calcium-fortified foods, for example, cereals, fruit juice, or tofu, are feasible items to get enough highly bioavailable calcium. The absorption of calcium is about 30% from dairy and fortified foods (e.g. orange juice, tofu, and soy drink) and nearly twice as high from certain green vegetables (e.g. bok choy, broccoli, and kale), but the degree of absorption varies because of adaptation and varying dietary composition. Depending on solubility, chemical form, and on other factors of the food, between 10 and 40% of dietary calcium is absorbed; for example, the percentage of absorption from calcium-fortified soy drinks and cow’s milk is similar. Mineral waters can also be a good source of absorbable calcium (16, 87).

Plant-based sources of calcium may be less bioavailable and, in turn, problematic for ensuring adequate calcium intake (7). Some foods contain compounds, such as oxalic acid and phytic acid, that bind calcium or otherwise interfere with calcium absorption. For instance, rhubarb, spinach, and walnuts are rich in oxalate, which forms sparingly soluble calcium oxalate. Among the foods high in phytic acid are fiber-containing whole-grain products and wheat bran, beans, seeds, nuts, and soy isolates. The extent to which these compounds affect calcium absorption varies (2, 16).

The aim of this review was to update the evidence relating to effects between calcium intake and health outcomes focusing mainly on SRs and meta-analyses. The quality of the studies is heterogeneous, not only in relation to age, sex, and lifestyle, but also to type of intervention, sources and doses, timing of supplementation, baseline calcium intake, or vitamin D concentration. The heterogeneity makes it difficult to interpret the results and provide single summary statements. Although there have been several RCTs, SRs, and meta-analyses evaluating the benefits of calcium intake on health outcomes, there is so far no convincing evidence that in healthy people, an intake of calcium above 1,000 mg a day is needed, and benefits of high dietary or supplemented calcium intake seem to remain minor. The recommended upper limit for calcium intake is 2,500 mg a day for adults, while the recommendations rarely present the lower tolerable limit, even long-term low calcium intake may cause health problems. In NNR2012, the lower intake level for calcium was 400 mg a day (33).

In general, the food pattern is more important than a single nutrient. Most nutrients act in all tissues, and inadequate intake impairs many body systems. Calcium works together with vitamin D, and the benefits will not develop if the intake of one or the other is suboptimal. Interdependencies among nutrients may well be a part of the explanation for the heterogeneity of results from different research centers and investigators.

Bone mass is ultimately determined by the genetic program as modified by current and past mechanical loading and limited or permitted by nutrition. The genetic potential of bone mass cannot be reached or maintained if dietary calcium intake and absorption is insufficient. Whenever absorbed calcium is insufficient to meet the demands of growth or the drain of cutaneous and excretory losses, resorption will be stimulated, and bone mass will be reduced.

There was no evidence that calcium intake would increase cardiovascular events. Regarding adverse events, dietary calcium intake seemed to be safe, while calcium supplements may cause, for example, gastrointestinal symptoms, such as constipation. Dietary calcium was not associated with kidney stones, while combined supplementation of calcium with vitamin D may increase the incidence of kidney stones.
